# The betaine content of sweat from adolescent females

**DOI:** 10.1186/1550-2783-7-3

**Published:** 2010-01-22

**Authors:** Shona S Craig, Stuart AS Craig, Matthew S Ganio, Carl M Maresh, Horrace Greg, Kerry-Ann da Costa, Steven H Zeisel

**Affiliations:** 1 Somers High School, Lincolndale, NY, USA; 2 Danisco A/S, Elmsford, NY, USA; 3 Human Performance Laboratory, Department of Kinesiology, University of Connecticut, Storrs, CT, USA; 4 Department of Nutrition, School of Public Health and School of Medicine, University of North Carolina, Chapel Hill, NC, USA

## Abstract

**Background:**

This study was developed to establish whether betaine was present in the sweat of females and to determine any correlations with other sweat components.

**Methods:**

Sweat patches were placed on eight trained adolescent Highland dancers (age = 13.6 ± 2.3 yr), who then participated in a dance class for 2 hours. Patches were removed, and the sweat recovered via centrifugation. The sweat was subsequently analyzed for betaine, choline, sodium, potassium, chloride, lactate, glucose, urea and ammonia.

**Results:**

Betaine was present in the sweat of all subjects (232 ± 84 μmol·L^-1^), which is higher than typically found in plasma. The concentration of several sweat components were correlated, in particular betaine with most other measured components.

**Conclusion:**

Betaine, an osmoprotectant and methyl donor, is a component of sweat that may be lost from the body in significant amounts.

## Background

Betaine is a methylamine that is widely distributed in nature where it is found in microorganisms, plants and animals. It is a significant component of many foods, including whole grains (e.g. wheat, rye), spinach, shellfish and beets [1], and low levels of dietary intake may increase disease risk [2-5]. Betaine is a trimethyl derivative of glycine that functions as an organic osmolyte to protect cells under stress (e.g. dehydration, high concentrations of electrolytes, urea and ammonia) and as a source of methyl groups for use in many key pathways via the methionine cycle [2]. Betaine accumulates in most tissues (e.g. liver, kidney, intestine, skin, muscle, etc.) [6], is non-perturbing to cellular metabolism, highly compatible with enzyme function, and stabilizes cellular metabolic function [2,7-14]. Betaine plays an important role in several aspects of human health and nutrition and recent studies show that ingestion of betaine may improve athletic performance [15-17].

Betaine concentration has been measured in many human tissues and fluids, including blood and urine, but has not been previously studied in sweat. Sweat can be considered a filtrate of plasma, cellular and interstitial fluid that contains electrolytes (e.g. potassium, sodium, and chloride), metabolic wastes (e.g. urea, ammonia and lactic acid), and various nutrients (e.g. vitamins and choline) [18-21]. The exact composition of sweat is dependent on several factors, including absorptive mechanisms in the sweat glands that may increase or decrease the concentration of solutes. We hypothesized that since betaine is a component of plasma and skin, it is also likely to be present in sweat. In addition, the above-mentioned protective role of betaine against electrolytes and metabolic wastes may extend to the sweat gland, duct and surrounding tissue. This study was conducted to determine whether betaine is a component of sweat that may be lost from the body during exercise.

## Methods

### Subjects

Eight trained female Scottish Highland dancers (10-17 yr) were recruited from the Stirling Highland Dance Company, Oakdale CT. The subjects trained regularly, and were actively competing in dance competitions. Subjects attended a briefing meeting before any experimentation to ensure an understanding of the testing parameters and the benefits/risks of the study. The subjects and parents signed a written informed consent statement. The study was part of the Somers High School (SHS) Science Research Program and the protocol was approved by the SHS IRB.

### Experimental Protocol

Sweat patches were prepared by placing two 2" × 2" gauze squares onto 4" × 4.5" adhesive film. Care was taken to minimize any cross-contamination. New disposable latex gloves were utilized for each subject. The skin on the lower back of the subjects was cleaned with gauze and distilled water, dried, and two patches were placed on both sides of the spine. The dancers then conducted a 2 hour class. The sweat patches were removed, placed in plastic 6-ml centrifuge tubes and stored on ice prior to centrifugation. The tubes were spun for 2 min at 1315 g in a benchtop centrifuge (Model 0151; Clay Adams, Parsippany, NJ). The patches were removed from the tubes, and the sweat (1-2 ml) at the bottom of the tubes was recovered. Each subject had two tubes from the two patches. The sweat from the two tubes was combined and stored frozen at -20°C prior to analysis.

### Measurements

Betaine, choline, and choline metabolites were determined in duplicate by liquid chromatography/electrospray ionization-isotope dilution mass spectrometry [22]. Lactate and glucose were determined in duplicate by enzymatic techniques (YSI 2300 Stat Plus, Yellow Springs, OH). Sodium, potassium and chloride were measured in duplicate using ion selective electrodes (Medica Easy Electrolytes, Medica Corp., Bedford, MA). Urea and ammonia were measured using a COBAS Mira Plus Analyzer (Roche Diagnostics, Indianapolis, IN) and Pointe Scientific (Canton, MI) reagent sets and standards. Instruments were calibrated using NIST certified standards.

### Statistics

Grubbs' test http://graphpad.com/quickcalcs/Grubbs1.cfm was used to determine outliers in data sets (alpha = 0.05). Pearson's correlation test (SigmaPlot v11, Systat Software Inc, San Jose, CA) and Passing-Bablok regression analysis (MedCalc, Mariakerke, Belgium) were conducted to compare data sets.

## Results

**Figure 1:**
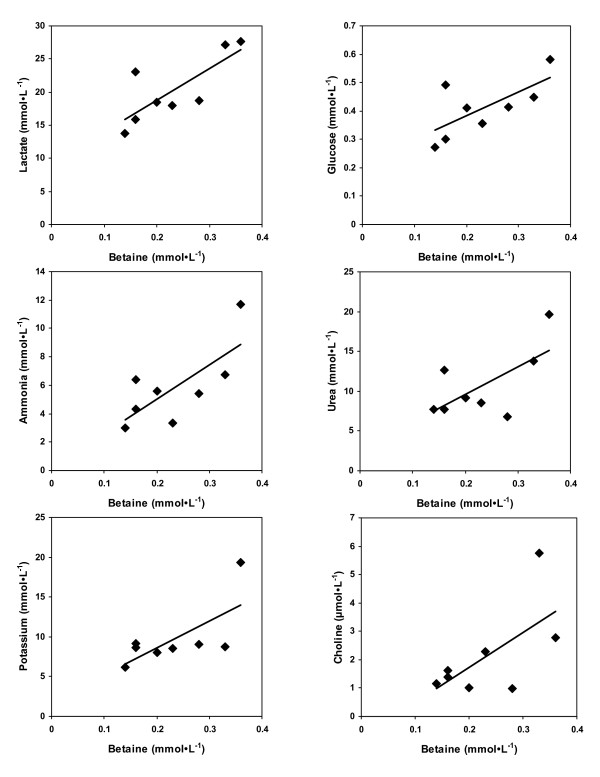
Correlations between betaine and other components of sweat

The measures of sweat composition are shown in Table 1. Phosphatidylcholine and sphingomyelin were also measured, but were not detected (data not shown). The mean betaine content was 232 ± 84 μmol·L^-1^. The other components of sweat were found at levels similar to that of previous studies [18,19,21]. Four data points were identified (*) as outliers via Grubb's test. Pearson's correlations test and Passing-Bablok regression analysis were conducted for the data in Table 1. Table 2 shows the Pearson's correlation coefficients between sweat components - values that show statistical significance (p < 0.05) or trends (p < 0.10) are in bold. Betaine is correlated with all components except sodium and chloride (Fig. 1). The non-parametric regression analysis (Passing-Bablok) gave similar results (not shown). None of the Pearson's correlations for potassium remain after removal of a data point (19.3 mmol·L^-1^) that is an outlier via Grubb's test (Table 1). Table 3 compares the content of sweat measured in this study with typical fasting levels published for plasma [18,23-26].

**Table 1:** Sweat composition of subjects

Subject	Betaine (μmol·L^-1^)	Choline (μmol·L^-1^)	Lactate (mmol·L^-1^)	Glucose (μmol·L^-1^)	Sodium (mmol·L^-1^)	Potassium (mmol·L^-1^)	Chloride (mmol·L^-1^)	Ammonia (mmol·L^-1^)	Urea (mmol·L^-1^)
1	363	2.77	27.6	582	37.9	19.3*	29.1	11.73*	19.68
2	160	1.38	15.7	302	46.7	8.62	34.6	4.31	7.69
3	332	5.75*	27.2	447	46.6	8.73	35.2	6.75	13.77
4	277	0.98	18.7	415	52.4	9.06	37.7	5.41	6.75
5	140	1.17	13.8	272	52.0	6.20	36.5	3.01	7.67
6	157	1.61	23.1	491	40.9	9.11	26.5	6.40	12.61
7	196	1.01	18.5	411	36.3	8.03	24.9	5.57	9.17
8	229	2.28	18.0	356	81.7*	8.59	57.6*	3.34	8.59
									
Average	232	2.12	20.4	410	49.3	9.7	35.3	5.81	10.74
SD	84	1.60	5.1	101	14.4	4.0	10.2	2.74	4.38

* Outlier via Grubb's Test (p < 0.05)

**Table 2:** Pearson's correlations (r) for sweat components

	Betaine	Choline	Lactate	Glucose	Sodium	Potassium	Chloride	Ammonia	Urea
Betaine	x	**+0.65^#^**	+0.78*	**+0.69^#^**	-0.08	**+0.70^#^**	+0.03	+0.73*	**+0.67^#^**
Choline		x	+0.72*	+0.36	+0.02	+0.21	+0.10	+0.36	+0.55
Lactate			x	+0.90*	-0.36	+0.67*	-0.31	+0.85*	+0.89*
Glucose				x	-0.45	+0.79*	-0.43	+0.92*	+0.86*
Sodium					x	-0.31	+0.99*	-0.57	-0.43
Potassium						x	-0.23	+0.92*	+0.85*
Chloride							x	-0.50	-0.37
Ammonia								x	+0.92*
Urea									x

*p < 0.05

^#^p < 0.10

**Table 3:** Solute contents of sweat compared with published fasting values for plasma [18,23-26]

	Sweat (S)	Plasma (P)
Betaine (μmol·L^-1^)	232	34.0
Choline (μmol·L^-1^)	2.1	14.5
Lactate (mmol·L^-1^)	20.4	0.7
Glucose (mmol·L^-1^)	0.41	4.9
Sodium (mmol·L^-1^)	49.3	141
Potassium (mmol·L^-1^)	9.7	4.1
Chloride (mmol·L^-1^)	35.3	105
Ammonia (mmol·L^-1^)	5.81	0.07
Urea (mmol·L^-1^)	10.74	5.7

We observed that betaine levels can drop if kept at room temperature for prolonged periods; therefore, it is important when collecting sweat samples to keep them in crushed ice until frozen. We speculate that enzyme or bacterial action might reduce betaine levels, but this requires further study. Also, preliminary results (not shown) suggest that betaine levels in sweat are higher after ingestion of betaine. Future work on the relationship between plasma and sweat levels is warranted.

## Discussion

This is the first study to determine the betaine content of sweat, and the average concentration in adolescent female sweat (232 ± 84 μmol·L^-1^) was found to be about 7 times higher than that typically found in female plasma (34 ± 11 μmol·L^-1^) [25]. The majority of constituents in sweat, such as sodium, chloride, glucose and choline, are more dilute than in the blood plasma or interstitial fluid [20]. However, some constituents are more concentrated in sweat, such as lactate, urea, ammonia, and potassium to a small extent. There are studies that support the concept of higher betaine concentrations in sweat versus plasma. Firstly, betaine is actively accumulated as an osmolyte in skin cells under osmotic and oxidative stress [12,27]. Also, there are higher betaine concentrations (expressed as μmol·L^-1 ^tissue water) in rat skin (males 412 ± 185 μmol·L^-1^; females 305 ± 153 μmol·L^-1^) compared to rat plasma (males 186 ± 43 μmol·L^-1^; females 101 ± 37 μmol·L^-1^) [6].

Mean dietary intake of betaine was recently estimated to be 100-200 mg/d [28,29]. Loss via urine averages about 10 mg/d [30]. Sweat rates are variable, but daily fluid requirements for sedentary to very active persons range from 2-4 L/d in temperate climates and from 4-10 L/d in hot climates [31]. Therefore, a range of 2-10 L/d sweat loss translates to a betaine loss of approximately 50-270 mg/d from the regional sweat data. These results suggest that betaine loss through sweat is greater than that lost through urine and may even exceed dietary intake in some cases. Collection of sweat using regional patches is convenient and useful for relative comparisons, but the concentration of sweat constituents tends to be higher compared to values using whole body washdown [32,33]. Therefore further work is required to accurately determine total body loss, perhaps under varied exercise conditions. In addition, it would be valuable to determine any correlation between dietary intakes, serum concentrations, sweat concentrations and level of physical activity.

The data showed several statistically significant correlations between sweat metabolites. Not surprisingly, the strongest correlation was between sodium and chloride. Betaine was correlated with all components except sodium and chloride (somewhat surprising given the known relationship between betaine accumulation and salt tolerance). The correlation between lactate and potassium agrees with the correlation found (+0.78) in a previous study [33] in males. Muscle contractions cause lactic acidosis and loss of intracellular potassium with accumulation of extracellular potassium [34]. Lactic acid acidification has been shown to counteract the effects of elevated potassium associated with muscle fatigue [35]. This may form the basis of a correlation. Betaine, lactate and glucose were all correlated with each other. Lactate and glucose are closely related via anaerobic metabolism. Also, a study showed that ingestion of betaine led to elevated serum lactate [15]. Although the current study was not designed to determine causation, betaine may accumulate to protect the sweat gland, duct and surrounding tissue from the deleterious effects of elevated concentrations of inorganic ions, urea, ammonia and possibly lactate - which are known to perturb cellular metabolism. Betaine protects the kidney from high concentrations of electrolytes and urea [2,36,37], prevents myosin structural change due to urea [9], and protects against ammonia toxicity of neurons [14]. This may relate to the correlations between betaine, ammonia, urea, lactate and potassium found here in sweat. Further research on the significance and reproducibility of these correlations is warranted.

In conclusion, betaine is a component of sweat. Betaine is an osmoprotectant, and we speculate that it protects the sweat gland against the deleterious effects of other sweat components. Further research is warranted, such as evaluation of male and/or older athletes, sweat collection via total body washdown [38], and determination of any correlation between type of exercise, plasma betaine levels, dietary intake of betaine, and sweat composition.

## Competing interests

Stuart Craig is employed by Danisco A/S, a manufacturer of betaine.

## Authors' contributions

SSC was the primary investigator, study design, data collection/analysis and manuscript draft. SASC conceived the study, supervised, statistical analysis, manuscript preparation. MSG, KAC supervised and sweat analysis. CMM, GH, SHZ participated in concept, design, coordination and helped draft the manuscript. All authors read and approved the final manuscript.

## References

[B1] Zeisel SH Mar MH Howe JC Holden JM Concentrations of choline-containing compounds and betaine in common foods J Nutr 2003 133 1302 1307 12730414 10.1093/jn/133.5.1302

[B2] Craig SAS Betaine in human nutrition Am J Clin Nutr 2004 80 539 549 15321791 10.1093/ajcn/80.3.539

[B3] Konstantinova SV Tell GS Vollset SE Nygard O Bleie O Ueland PM Divergent associations of plasma choline and betaine with components of metabolic syndrome in middle age and elderly men and women J Nutr 2008 138 914 920 18424601 10.1093/jn/138.5.914

[B4] Cho E Willett WC Colditz GA Fuchs CS Wu K Chan AT Zeisel SH Giovannucci EL Dietary Choline and Betaine and the Risk of Distal Colorectal Adenoma in Women J Natl Cancer Inst 2007 1224 1231 17686825 10.1093/jnci/djm082 PMC2441932

[B5] Shaw GM Carmichael SL Yang W Selvin S Schaffer DM Periconceptional dietary intake of choline and betaine and neural tube defects in offspring Am J Epidemiol 2004 160 102 109 10.1093/aje/kwh187 15234930

[B6] Slow S Lever M Chambers ST George PM Plasma dependent and independent accumulation of betaine in male and female rat tissues Physiol Res 2009 58 403 410 18637704 10.33549/physiolres.931569

[B7] Yancey PH Clark ME Hand SC Bowlus RD Somero GN Living with water stress: evolution of osmolyte systems Science 1982 217 1214 1222 10.1126/science.7112124 7112124

[B8] Olsen SN Ramlov H Westh P Effects of osmolytes on hexokinase kinetics combined with macromolecular crowding Test of the osmolyte compatibility hypothesis towards crowded systems Comp Biochem Physiol A Mol Integr Physiol 2007 148 339 345 10.1016/j.cbpa.2007.05.009 17581767

[B9] Ortiz-Costa S Sorenson MM Sola-Penna M Betaine protects urea-induced denaturation of myosin subfragment-1 FEBS Journal 2008 13 3388 3396 10.1111/j.1742-4658.2008.06487.x 18494798

[B10] Wray S Wilkie D The relationship between plasma urea levels and some muscle trimethylamine levels in Xenopus laevis: a 31P and 14N nuclear magnetic resonance study J Exp Biol 1995 198 373 378 7699312 10.1242/jeb.198.2.373

[B11] Viennet C Bride J Morel B Bodeau C Humbert P Glycine betaine stimulates human skin fibroblasts growth and collagen production in culture J Invest Dermatol 2002 118 1099

[B12] Warskulat U Reinen A Grether-Beck S Krutmann J Haussinger D The osmolyte strategy of normal human keratinocytes in maintaining cell homeostasis J Invest Dermatol 2004 123 516 521 10.1111/j.0022-202X.2004.23313.x 15304091

[B13] Coelho-Sampaio T Ferreira ST Castro EJ Junior Vieyra A Betaine counteracts urea-induced conformational changes and uncoupling of the human erythrocyte Ca2+ pump Eur J Biochem 1994 221 1103 1110 10.1111/j.1432-1033.1994.tb18830.x 8181468

[B14] Minana M Hermenegildo C Llsansola M Montoliu C Grisolia S Felipo V Carnitine and choline derivatives containing a trimethylamine group prevent ammonia toxicity in mice and glutamate toxicity in primary cultures of neurons J Pharmacol Exp Ther 1996 279 194 199 8858993

[B15] Armstrong LE Casa DJ Roti MW Lee EC Craig SA Sutherland JW Fiala KA Maresh CM Influence of betaine consumption on strenuous running and sprinting in a hot environment J Strength Cond Res 2008 22 851 860 18438230 10.1519/JSC.0b013e31816a6efb

[B16] Maresh CM Farrell MJ Kraemer WJ Yamamoto LM Lee EC Armstrong LE Hatfield DL Sokmen B Dias JC Spiering BA The Effects of Betaine Supplementation on Strength and Power Performance Med Sci Sports Exerc 2007 39 S101

[B17] Hoffman J Ratamess N Kang J Rashti S Faigenbaum A Effect of Betaine Supplementation on Power Performance and Fatigue Journal of the International Society of Sports Nutrition 2009 6 7 17 10.1186/1550-2783-6-7 19250531 PMC2651845

[B18] Meyer F Laitano O Bar-Or O McDougall D Heingenhauser GJ Effect of age and gender on sweat lactate and ammonia concentrations during exercise in the heat Braz J Med Biol Res 2007 40 135 143 17225006 10.1590/s0100-879x2007000100017

[B19] Huang CT Chen ML Huang LL Mao IF Uric acid and urea in human sweat Chin J Physiol 2002 45 109 115 12817713

[B20] Mickelsen O Keys A The composition of sweat, with special reference to the vitamins J Biol Chem 1943 149 479 490

[B21] Johnson BC Hamilton TS Mitchell HH The effect of choline intake and environmental temperature on the excretion of choline from the human body J Biol Chem 1945 159 5 9

[B22] Koc H Mar MH Ranasinghe A Swenberg JA Zeisel SH Quantitation of choline and its metabolites in tissues and foods by liquid chromatography/electrospray ionization-isotope dilution mass spectrometry Anal Chem 2002 74 4734 4740 10.1021/ac025624x 12349977

[B23] FNB Dietary reference intakes for water, potassium, sodium, chloride, and sulfate 2004 Washington DC: The National Acadamies Press

[B24] Tirosh A Shai I Tekes-Manova D Israeli E Pereg D Shochat T Kochba I Rudich A the Israeli Diabetes Research Group Normal Fasting Plasma Glucose Levels and Type 2 Diabetes in Young Men N Engl J Med 2005 353 1454 1462 10.1056/NEJMoa050080 16207847

[B25] Lever M Sizeland PC Bason LM Hayman CM Chambers ST Glycine betaine and proline betaine in human blood and urine Biochim Biophys Acta 1994 1200 259 264 8068711 10.1016/0304-4165(94)90165-1

[B26] von Allworden HN Horn S Kahl J Feldheim W The influence of lecithin on plasma choline concentrations in triathletes and adolescent runners during exercise Eur J Appl Physiol Occup Physiol 1993 67 87 91 10.1007/BF00377711 8375373

[B27] Warskulat U Brookmann S Reinen A Haussinger D Ultraviolet B radiation induces cell shrinkage and increases osmolyte transporter mRNA expression and osmolyte uptake in HaCaT keratinocytes Biol Chem 2007 388 1345 1352 10.1515/BC.2007.140 18020950

[B28] Bidulescu A Chambless L Siega-Riz AM Zeisel S Heiss G Repeatability and measurement error in the assessment of choline and betaine dietary intake: the Atherosclerosis Risk in Communities (ARIC) Study Nutrition Journal 2009 8 14 10.1186/1475-2891-8-14 19232103 PMC2654540

[B29] Cho E Zeisel SH Jacques P Selhub J Dougherty L Colditz GA Willett WC Dietary choline and betaine assessed by food-frequency questionnaire in relation to plasma total homocysteine concentration in the Framingham Offspring Study Am J Clin Nutr 2006 83 905 911 16600945 10.1093/ajcn/83.4.905 PMC2430728

[B30] Lever M Atkinson W Sizeland PC Chambers ST George PM Inter- and intra-individual variations in normal urinary glycine betaine excretion Clin Biochem 2007 40 447 453 10.1016/j.clinbiochem.2006.10.029 17335790

[B31] Sawka MN Montain SJ Fluid and electrolyte supplementation for exercise heat stress Am J Clin Nutr 2000 72 564S 572 10919961 10.1093/ajcn/72.2.564S

[B32] Palacios C Wigertz K Weaver CM Comparison of 24 hour whole body versus patch tests for estimating body surface electrolyte losses Int J Sport Nutr Exerc Metab 2003 13 479 488 14967871 10.1123/ijsnem.13.4.479

[B33] Patterson MJ Galloway SD Nimmo MA Variations in regional sweat composition in normal human males Exp Physiol 2000 85 869 875 10.1111/j.1469-445X.2000.02058.x 11187982

[B34] Brooks GA Lactate doesn't necessarily cause fatigue: why are we surprised? J Physiol 2001 536 1 10.1111/j.1469-7793.2001.t01-1-00001.x 11579151 PMC2278833

[B35] Nielsen OB de Paoli F Overgaard K Protective effects of lactic acid on force production in rat skeletal muscle J Physiol 2001 536 161 166 10.1111/j.1469-7793.2001.t01-1-00161.x 11579166 PMC2278832

[B36] Horio M Ito A Matsuoka Y Moriyama T Orita Y Takenaka M Imai E Apoptosis induced by hypertonicity in Madin Darley canine kidney cells: protective effect of betaine Nephrol Dial Transplant 2001 16 483 490 10.1093/ndt/16.3.483 11239020

[B37] Yancey PH Burg MB Counteracting effects of urea and betaine in mammalian cells in culture Am J Physiol 1990 258 R198 204 2301632 10.1152/ajpregu.1990.258.1.R198

[B38] Armstrong LE Casa DJ Methods to Evaluate Electrolyte and Water Turnover of Athletes Athletic Training & Sports Health Care 2009 1 169 179

